# RNA Interference towards the Potato Psyllid, *Bactericera cockerelli*, Is Induced in Plants Infected with Recombinant *Tobacco mosaic virus* (TMV)

**DOI:** 10.1371/journal.pone.0066050

**Published:** 2013-06-18

**Authors:** Hada Wuriyanghan, Bryce W. Falk

**Affiliations:** 1 Department of Plant Pathology, University of California Davis, Davis, California, United States of America; 2 Life Science College, Inner Mongolia University, Hohhot, Inner Mongolia, People’s Republic of China; Ghent University, Belgium

## Abstract

The potato/tomato psyllid, *Bactericera cockerelli* (*B. cockerelli*), is an important plant pest and the vector of the phloem-limited bacterium *Candidatus* Liberibacter psyllaurous (solanacearum), which is associated with the zebra chip disease of potatoes. Previously, we reported induction of RNA interference effects in *B. cockerelli* via *in vitro*-prepared dsRNA/siRNAs after intrathoracic injection, and after feeding of artificial diets containing these effector RNAs. In order to deliver RNAi effectors via plant hosts and to rapidly identify effective target sequences in plant-feeding *B. cockerelli*, here we developed a plant virus vector-based *in planta* system for evaluating candidate sequences. We show that recombinant *Tobacco mosaic virus* (TMV) containing *B. cockerelli* sequences can efficiently infect and generate small interfering RNAs in tomato (*Solanum lycopersicum*), tomatillo (*Physalis philadelphica*) and tobacco (*Nicotiana tabacum*) plants, and more importantly delivery of interfering sequences via TMV induces RNAi effects, as measured by actin and V-ATPase mRNA reductions, in *B. cockerelli* feeding on these plants. RNAi effects were primarily detected in the *B. cockerelli* guts. In contrast to our results with TMV, recombinant *Potato virus X* (PVX) and *Tobacco rattle virus* (TRV) did not give robust infections in all plants and did not induce detectable RNAi effects in *B. cockerelli*. The greatest RNA interference effects were observed when *B. cockerelli* nymphs were allowed to feed on leaf discs collected from inoculated or lower expanded leaves from corresponding TMV-infected plants. Tomatillo plants infected with recombinant TMV containing *B. cockerelli actin* or *V-ATPase* sequences also showed phenotypic effects resulting in decreased *B. cockerelli* progeny production as compared to plants infected by recombinant TMV containing *GFP*. These results showed that RNAi effects can be achieved in plants against the phloem feeder, *B. cockerelli*, and the TMV-plant system will provide a faster and more convenient method for screening of suitable RNAi target sequences *in planta*.

## Introduction

RNA interference (RNAi) is a natural gene regulation and antiviral defense system of eukaryotes. Double-stranded RNAs (dsRNA), which can be produced in the nucleus by endogenous transcription, taken up by the cells from exogenous sources, or result from virus (particularly RNA virus) infection, are cleaved by the RNase III family endoribonuclease Dicer to yield 21–24 nucleotide small interfering RNAs (siRNAs). The RNA-induced silencing complex (RISC) incorporates one strand (the guide strand) from the siRNAs and this serves to sequester the complementary sequence (by base-pairing) within the specific target RNA. The Argonaute protein, which is a component of RISC, then degrades the target RNA. RNAi can therefore be manipulated to suppress endogenous gene expression through siRNA-mediated degradation of mRNA transcripts [1], [2], and is a powerful reverse genetics tool to study gene function in a variety of organisms. RNAi has become an intensely studied research area with great potential for fundamental and practical biology. For example, in plants, RNAi-based technologies have been used for generation of plants which are resistant toward bacterial, fungal, nematode or virus-induced plant diseases [3]–[6], and even showed promise for targeting parasitic weeds such as *Triphysaria versicolor* [7].

Recently, RNAi technology has also been proposed as a potentially useful tool for plant-associated insect control. Most insect RNAi studies reported to date have used *in vitro*-transcribed dsRNAs as effectors to induce RNAi activity in recipient insects [8]. The dsRNA effectors have been delivered either via intrathoracic injection or via oral feeding. Injection into hemocoel is a routine way to introduce dsRNAs in many insect species, but is artificial from a practical sense [9]–[13]. Successful RNAi effects via oral feeding of *in vitro* synthesized dsRNAs have also been demonstrated in insects of several orders, supporting the feasibility of providing RNAi inducers in the target insects’ diet [14]–[17], even via transgenic plants [18]–[21]. Even if stably transformed plants are the ultimate step toward practical application of RNAi for insect control, development of appropriate transgenic plants is a laborious, time consuming and relatively expensive process. Therefore, an experimental system amenable to rapidly screen RNAi effectors and identify optimal target RNAs would be highly desirable. In this regard, recombinant plant viruses could be a more efficient and rapid alternative for evaluating effective interfering and target sequences in plants.

Plant RNA viruses are powerful inducers and targets of RNAi responses in plants. RNA plant viruses replicate in the cell cytoplasm via double-stranded RNA intermediates, and plants respond to virus infections by RNA interference thereby generating large amounts of virus-specific siRNAs. Thus, RNA plant virus infections yield two types of RNAi inducers, double-stranded RNAs and siRNAs, and if the virus contains a foreign sequence, double-stranded RNA and siRNA forms of this sequence also will be generated. Recombinant plant viruses have been exploited as powerful tools to induce RNAi effects directly in their host plants [22], and recently, recombinant plant viruses have also been employed to obtain trans-specific RNAi effects during the interactions between host plants and their biotrophic plant pathogens. Expression of sequences of the wheat stripe rust fungus *Puccinia striiformis* using *Barley stripe mosaic virus* (BSMV) system in infected wheat led to reduced expression of the corresponding mRNAs in the target *P. striiformis* [23]. Valentine et al have also demonstrated the feasibility of using a recombinant plant virus to target and induce RNAi effects in plant parasitic nematodes [24]. Most recently, *Tobacco rattle virus* (TRV) expression of antisense fragments from a chewing insect *Manduca sexta* in *Nicotiana attenuata* plants specifically silenced three midgut-expressed *MsCYPs* genes when larvae fed on these plants [25]. So far plant viruses have not been used to target xylem or phloem-feeding hemipterans, many of which are important plant pests and/or vectors of specific plant pathogens.

The potato/tomato psyllid, *Bactericera cockerelli* (*B. cockerelli*), is a serious pest of potato and tomato plants. *B. cockerelli* is associated with plant diseases including psyllid yellows of tomatoes [26] and zebra chip disease of potatoes [27], and is the vector of the phloem-limited bacterium, *Candidatus* Liberibacter psyllaurous (solanacearum). Previously, we reported that RNA interference effects in *B. cockerelli* were inducible via injection and oral feeding of *in vitro*-prepared dsRNAs and siRNAs, demonstrating the existence of RNAi machinery in this psyllid, and suggesting the possibility of using RNAi technology to help control *B. cockerelli* and/or associated plant diseases [28]. In the present study, we demonstrate for the first time plant virus-induced RNAi effects in the hemipteran phloem feeder, *B. cockerelli*. Our hypothesis is that the virus-infected plant will generate siRNAs in defense towards the infecting virus and the recombinant (*B. cockerelli*) cDNA sequence it carries [29]. The recombinant virus causes a systemic infection in the plant, interacting with many cell types including those in the phloem, and the phloem also is an effective conduit for transport of small and even large RNAs [30], [31], and thus will provide a continuous source of silencing RNAs for the phloem-feeding insects such as *B. cockerelli*. We show that *B. cockerelli* fed on plants infected with recombinant *Tobacco mosaic virus* (TMV) containing *B. cockerelli BC- ATPase* and/or *BC-Actin* sequences exhibit RNAi effects as measured by reduction in target mRNA levels and the number of progeny produced on these plants. These data suggest that plant virus expression systems can allow for rapid, efficient screening of *B. cockerelli* candidate gene targets for RNAi efficiency *in planta.*

## Materials and Methods

### 1. Insect Culture

The *B. cockerelli* used in this study were originally obtained from Dr. T. D. Paine’s laboratory, University of California, Riverside [26]. *B. cockerelli* were reared and maintained on tomato plants (*Solanum lycopersicum,* Early Pak 7) in mesh cages within growth chambers at 25°C and 70% humidity under 14∶10 (light: dark) photoperiod. Synchronous nymphs and teneral adult *B. cockerelli* were used for the feeding experiments as described [28].

### 2. Recombinant Virus Vector Construction

We used three plant viruses here, which are *Tobacco mosaic virus* (TMV), *Tobacco rattle virus* (TRV) and *Potato virus X* (PVX). In all of the following constructs for *BC-ATPase* (JN108000.1), 386 bp PCR fragments from the *BC-ATPase* gene were amplified using the sense primer 5′-CTAATACGACTCACTATAGG*GCGGCCGC*TTGTGCTGGACCCTACCATT-3′ and antisense primer 5′- CTAATACGACTCACTATAGG*GCGGCCGC*CAATCATGCCTCCAATGATG-3′ where the restriction endonuclease site NotI is underlined. The NotI-digested *BC-ATPase* PCR amplicon was ligated into the NotI-digested TMV vector, pJL36 [32]. The multiple cloning site of PVX (pP2C2S) [33] was modified as we inserted a fragment of 50 bp which contained the NotI endonuclease site. The NotI-digested *BC-ATPase* PCR amplicon was cloned into the NotI site of this modified vector. TRV vectors including TRV1 and TRV2 (pYL279) were kindly provided by Dr. Dinesh Kumar [34]. As pYL279 is a TRV2 gateway vector, the *BC-ATPase* PCR amplicon was first cloned into the pCR®8/GW/TOPO® TA vector, and then was recombined into pYL279 vector using Gateway® LR Clonase® enzyme mix (Invitrogen cat. NO. 11791-020). *BC-Actin* (JN107999.1) was cloned into TMV by amplifying a 364 bp amplicon using the primers 5′-CTAATACGACTCACTATAGG*GCGGCCGC*CTCCCTGTACGCCTCTGGT -3′ and 5′- CTAATACGACTCACTATAGG*GCGGCCGC*GCAGCTCGTAGCTCTTCTCC-3′, where the NotI site is underlined, and ligated into NotI-digested pJL36. Control pJL24 (TMV-GFP) plasmid, which contains the GFP open reading frame, was obtained from Dr. John Lindbo [32]. The pYL36 (TRV-GFP) vector, a TRV2 plasmid containing the GFP open reading frame, was provided by Dr. Dinesh Kumar [34].

### 3. Virus Infection and *B. cockerelli* Bioassays

*N. benthamiana*, tomato (*Solanum lycopersicum*), tomatillo (*Physalis philadelphica*) and Turkish tobacco (*Nicotiana tabacum*) plants were used for virus infection and/or *B. cockerelli* feeding experiments. Plants were grown in standard greenhouse conditions and virus inoculations were performed as follows: *Agrobacterium tumefaciens* cells, GV3101, were transformed with the above plasmids for TMV and TRV (TRV1 and TRV2 separately). *A. tumefaciens* cells were grown in liquid culture and used for leaf infiltration of *N. benthamiana* plants (3–4 leaf stage) as described [32]. Plasmids for PVX were purified from *E. coli*, linearized with SpeI restriction endonuclease, transcribed *in vitro* using the mMESSAGE mMACHINE® T7 transcription Kit (Ambion, catalog NO. AM1344) as described before [35]. Five uL of the transcribed RNA was mixed with 45 uL of sterile FES buffer (1% sodium pyrophosphate, 1% bentonite, 1% celite in 0.1 M glycine, 0.06 M dibasic potassium phosphate) and inoculated onto leaves of *N. benthamiana* plants by rub inoculation. After systemic infections developed (14 days post inoculation), leaves were collected and used as inoculum for subsequent inoculations into tomato, tomatillo and Turkish tobacco plants as described [36]. In all cases virus-infected plants were tested by RT-PCR analysis to ensure that recombinant viruses retained the inserted sequences. Recombinant TMV, TRV and PVX engineered to express the green fluorescent protein (GFP) were used as controls. GFP protein expression was visualized by Ultraviolet (UV) light.

*B. cockerelli* RNAi assays were done using the plants 14 days post virus inoculation. Three approaches were used initially. These were using plastic cylindrical sleeve cages to confine teneral adult *B. cockerelli* onto whole plants; using mesh bags to contain *B. cockerelli* on inoculated or lower expanded leaves; and by placing *B. cockerelli* nymphs onto leaf discs cut from inoculated or lower expanded leaves and placed within 12-well tissue culture plates containing a small amount of MS medium (Fig. S1) [37]. For tobacco, inoculated leaves were used for feeding assays whereas for tomato and tomatillo, lower expanded leaves were used unless stated otherwise.

Quantification of *BC-ATPase* mRNAs was carried out 3-days after feeding teneral adult *B. cockerelli* on whole plants (Fig. S1 a), or nymphs on leaf discs (Fig. S1 c). *B.cockerelli* were collected and used individually for RNA isolation. To analyze *B. cockerelli* tissue-specific silencing effects, teneral adult *B. cockerelli* were fed on lower expanded leaves of tomatillo plants in mesh cages (Fig. S1 b) for 3 days. A group of ∼100 *B. cockerelli* were dissected and used for gut and abdomen RNA isolation.

*B. cockerelli* mortality and fecundity assays were performed using 20 teneral adults which were fed on TMV-infected tomatillo plants within cylindrical cages. Surviving *B. cockerelli* were counted on the 7^th^ day and removed from the cage. Plants were maintained and the numbers of nymphs developing on these plants were determined 14 days later. The experiments were repeated three times and five biological replicates were included in each experiment.

### 4. Northern Hybridization Analysis and qRT-PCR Quantification of RNAs in Plants and *B. cockerelli*

Northern hybridization analysis was used to identify virus-specific RNAs acquired by feeding *B. cockerelli*. *B. cockerelli* nymphs were fed on *GFP* recombinant virus-infected leaf discs for 1 day, and RNA was isolated from a pool of ∼60 nymphs. Northern hybridization analysis was performed using 1 µg of plant RNA or 10 µg of total RNAs of *B. cockerelli*. ^32^P-UTP-labeled negative strand *GFP* transcripts were used as the probe to detect virus RNAs. Northern blot analysis was performed as described previously [28].

We also used *GFP* sequence-specific quantitative real-time PCR to quantify *GFP* recombinant virus RNAs within *B. cockerelli* after feeding on TMV-GFP infected tomatillo plants. cDNA was synthesized using three different primers including random hexamers (Invitrogen) which will serve as primers for both positive and negative strand virus RNAs, pJL36 Left primer (5′-AGATCTTACAGTATCACTACTCC-3′) which specifically primes on the negative strand of TMV RNA, and pJL36 right primer (5′- GTACGCACCACGTGTGATTACGG-3′) which specifically primes on the positive strand of TMV genomic or subgenomic RNAs. The cDNAs were used for quantification by using *GFP*-specific qPCR primers 5′- CCTGTCCTTTTACCAGACAACCA-3′ and 5′- CACGCTTTTCGTTGGGATCT-3′, and the quantification was normalized against *B. cockerelli BC-Actin* mRNA abundance using the primers 5′- AGAGAGAAGATGACCCAGATCATGT -3′ and 5′- GCAGCTCGTAGCTCTTCTCC -3′.

### 5. *B. cockerelli* RNA Isolation, cDNA Synthesis and Quantitative Real Time PCR (qRT-PCR) for mRNA Abundance

Teneral adult or nymph *B. cockerelli* were fed on the virus-infected plants described above. Total RNAs were extracted from individual *B. cockerelli* or specific tissues dissected from pooled insects using Trizol. *B. cockerelli* dissection, cDNA synthesis and qPCR were described previously [28]. Target mRNAs were quantified by quantitative real-time PCR using Fast SYBR Green Master Mix (Applied Biosystems). In order to avoid RT-PCR artifacts resulting from the input RNA expressed by the virus vector, qRT-PCR primers for *BC-ATPase* and *BC-Actin* amplification were designed to detect target mRNAs by amplifying sequences that lay outside of the sequences contained within the virus vectors. For *BC-ATPase* mRNA analysis, *BC-Actin* mRNA abundance was used as an internal control for normalization after validation. For *BC-Actin* mRNA analysis, *BC-rRNA* mRNA abundance was used as an internal control for normalization after validation. GFP expressing plants were used as a control treatment for each virus infection and feeding experiment. The relative mRNA data were analyzed using the relative 2^−ΔΔCT^ method [38]. Briefly, the ΔCT value for individual samples was obtained by subtracting average Ct (cycle threshold) value for the internal control from average Ct value for the target transcript. One of the samples in the control groups, the *B. cockerelli* fed on GFP-construct- infected plants, was selected as a reference sample. The ΔΔCT value for individual samples was obtained by subtracting ΔCT value for the reference sample from the ΔCT value of the test individual sample. The relative abundance of target transcript in comparison to internal control level in each sample is represented by the 2-ΔΔCT value. The average value and standard error value (SE value) of the 2^−ΔΔCT^ value in treatment and control groups were calculated separately, and the statistical analyses were performed between these two groups using the Bonferroni t-test.

### 6. Small RNA Analysis

RNA hybridization analysis was used to determine if sequence-specific siRNAs were present in the plants inoculated with the recombinant viruses containing the *GFP*, *BC-ATPase* or *BC-Actin* sequences. Small RNAs were extracted from inoculated plants using the PEG-NaCl method. Briefly, total RNA was isolated from the plant samples using Trizol and large RNAs were precipitated using 1 M NaCl and 10% PEG8000. Small RNAs were recovered from the remaining supernatant using 3X volumes of cold ethanol. Small RNA hybridization was performed according to the methods described previously [28].

## Results

### 1. Evaluation of Different Recombinant Plant Viruses for Infection Efficiency and Small RNA Accumulation in Plants

**Figure 1:**
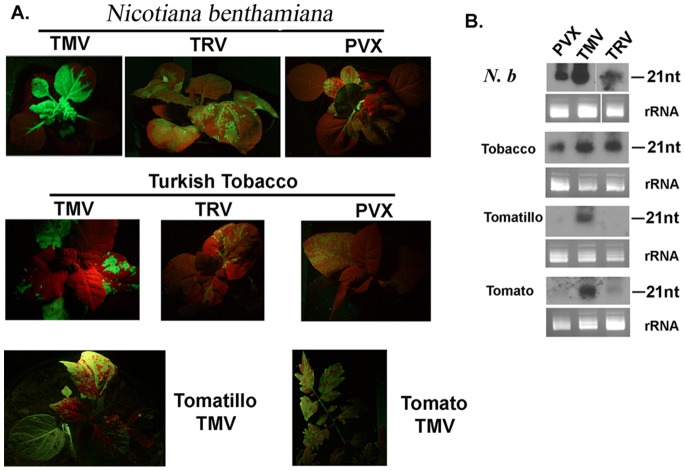
Infection of four different plant hosts with various virus vectors expressing GFP. (A), *N. benthamiana* (3–4 leaf stage), Turkish tobacco (4-leaf stage), tomatillo (cotyledons), and tomato (cotyledons) were inoculated by rub inoculation. Tomato plants infected with TMV-GFP were photographed under UV light to visualize GFP expression at 21 days post inoculation, whereas other plants (*N. benthamiana*, tomatillo, Turkish tobacco) were examined at 14 days post inoculation. The virus inocula were prepared in *N. benthamiana* (3–4 leaf stage) that were agro-infiltrated with TMV-GFP or TRV-GFP vectors using *Agrobacterium tumefaciens* GV3101, or rub-inoculated with *in vitro*-generated PVX-GFP transcripts in FES buffer. (B), Northern blot analysis showed the accumulation of ∼21 nt siRNAs in virus-infected plants. One µg of total small RNA was separated on a 15% PAGE gel containing 8 M Urea and transferred to a nylon membrane. ^32^P-UTP-labeled negative strand *GFP* RNA was used to probe the blot. MicroRNA markers were electrophoresed on the same gel. The migration of the 21 nt standard is indicated to the right of each blot. Below each blot are shown loading controls, ethidium bromide stained rRNAs.

In order to rapidly and efficiently synthesize the dsRNA/siRNA in planta, virus induced gene silencing (VIGS) vectors including TMV, PVX and TRV were tested in different plant species such as tomato, tomatillo and Turkish tobacco plants as they are all favorable hosts for *B. cockerelli*. We first compared various plant:virus combinations and evaluated both GFP expression and siRNA accumulation within infected plants. All virus-infected plants developed mild symptoms of virus infection, but were very suitable for our experiments here. We used GFP expression to compare different viruses as GFP protein can be easily visualized under UV light illumination. *N. benthamiana* plants were analyzed for expression of GFP protein and accumulation of *GFP* sequence specific siRNAs at 14 days after infection (Fig. 1). The *N. benthamiana* inocula were then used to infect tomato, tomatillo and tobacco plants. TMV-GFP vector was able to infect all of these three plants and produce high levels of *GFP* specific siRNAs (Fig. 1). By contrast, PVX and TRV only readily infected Turkish tobacco plants and showed *GFP* specific siRNAs at 14 days after infection. For tomato and tomatillo plants, cotyledons were used for inoculation and GFP expression was observed in lower fully expanded leaves at 3 and 2 weeks, respectively, post inoculation. Therefore, in the following *B. cockerelli* feeding experiments, the lower expanded leaves of tomatillo or tomato plants were used for mesh bag cage and leaf disc feeding experiments. For tobacco plants, infected leaves were used for feeding experiments as the inoculations were performed on 4-leaf stage plants and GFP expression was abundant on the same inoculated leaves. We used TMV/tomato, TMV/tomatillo, TMV/tobacco, TRV/tobacco and PVX/tobacco systems to test RNA interference efficiency in the following experiments.

### 2. Assessment of Different Plant:Virus Expression Systems and Feeding Protocols for RNAi Efficacy using *BC-ATPase* as the Target mRNA

**Figure 2:**
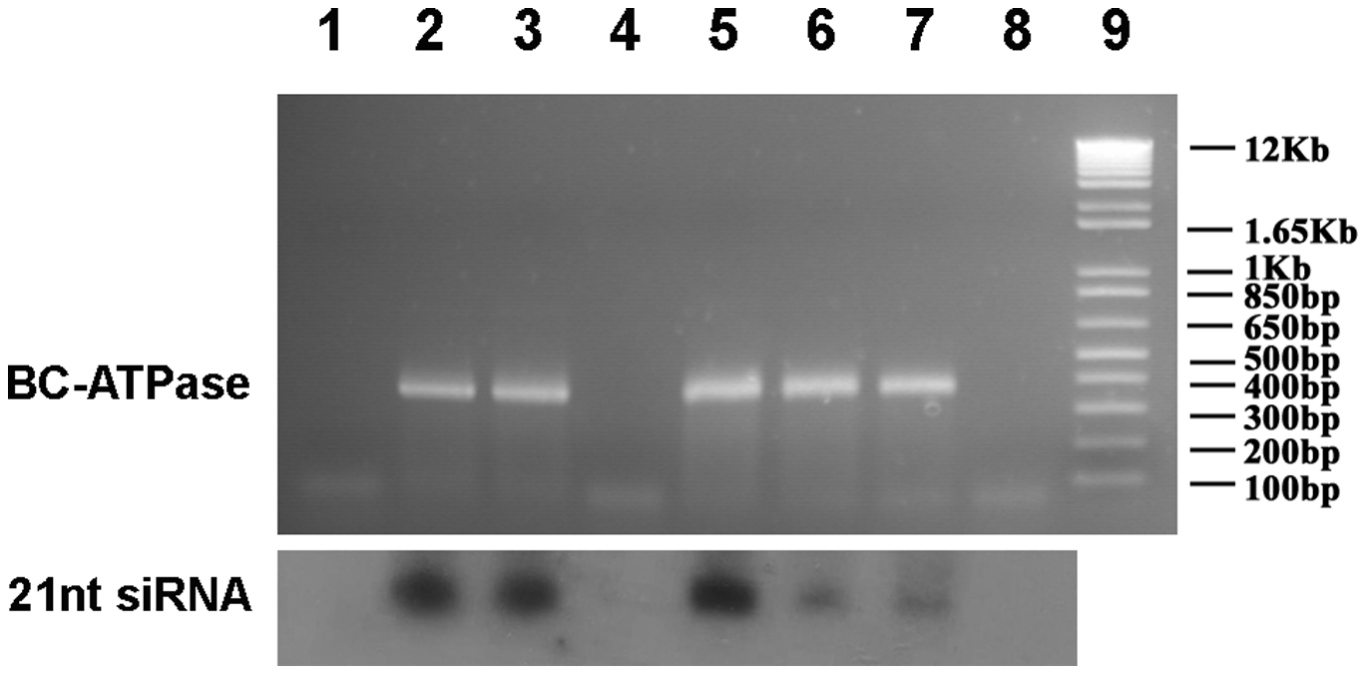
Detection of virus infection and accumulation of *BC-ATPase* siRNAs in plants. Plants were inoculated with various recombinant viruses carrying a *BC-ATPase* gene fragment. At 14 days post inoculation, total and small RNAs were isolated from infected plants and subjected to RT-PCR and siRNA northern blot analyses, respectively. Top panel: total RNA was reverse-transcribed using random hexamer primers and the cDNA was used as template for PCR by using *BC-ATPase* specific primers. The 386 bp product is visible in lanes 2, 3, 5, 6 and 7. Bottom panel: 1 µg small RNAs were separated on a 15% polyacrylamide gel containing 8 M Urea and transferred to a nylon membrane. ^32^P-UTP-labeled negative strand *BC-ATPase* RNA was used to probe the blot. Lane 1: control uninoculated tomato; Lane 2: tomato infected with TMV-ATPase; Lane 3: tomatillo infected with TMV-ATPase; Lane 4: control uninoculated tomatillo plant; Lane 5: tobacco plant infected with TMV-ATPase; Lane 6: tobacco plant infected with PVX-ATPase; Lane 7: tobacco plant infected with TRV-ATPase; Lane 8: control uninoculated tobacco plant; Lane 9: 1 kb Plus DNA ladder.

Previously, we screened several sequences for RNAi efficiency in *B. cockerelli* via dsRNA/siRNA injection and feeding assays. One of the sequences, *BC-ATPase,* showed consistent target mRNA down-regulation/degradation after both injection and feeding delivery of both dsRNAs and RNAaseIII-digestion generated siRNAs [28]. Here we asked whether RNA interference activity of this same sequence could be achieved via *in planta* virus expression. We used the TMV/tomato, TMV/tomatillo, TMV/tobacco, TRV/tobacco and PVX/tobacco expression systems. RT-PCR analysis showed that the *BC-ATPase* sequence was produced via the respective viruses in plants, and small RNA northern hybridization analysis showed accumulation of *BC-ATPase* siRNAs (Fig. 2). In initial experiments, we tested whole plant feeding where the teneral adult *B. cockerelli* were confined on whole plants in a cylindrical cage (Fig. S1 a). None of 6 feeding experiments showed significant reduction in *BC-ATPase* mRNA steady-state levels after *B. cockerelli* fed on TMV-ATPase*-*infected tomatillo plants compared with those fed on the control TMV-GFP plants. Similarly, with the tomato plant-TMV system only 1 out of 4 feeding experiments on plants showed a reduction (Table 1). We reasoned that the inconsistency of whole plant feeding method might be due to the disparity and non-uniformity of *in planta* virus spread and source-sink effects of phloem transport, therefore giving dis-similar expression/accumulation of interfering RNAs in different parts of the plants and then for the *B.cockerelli* feeding on these plant parts. Therefore, we next performed the feeding experiments with a leaf-disc method where *B. cockerelli* nymphs were fed on leaf discs cut from lower expanded leaves which were shown to express higher amounts of insert sequences (Fig. S1 c). *B. cockerelli* nymphs were used as they are less mobile than adults and also more active feeders (data shown later). For tomatillo plants, 3 out of 4 experiments showed a significant reduction of *BC-ATPase* mRNA accumulation after feeding on leaf discs of TMV-ATPase-inoculated plants compared with the control TMV-GFP plants (Table 1). Using the same leaf disc-nymph system, we detected significantly less of the *BC-ATPase* mRNA in 2 out of 3 experiments both for *B. cockerelli* feeding on leaf discs from TMV-ATPase inoculated tomato as well as Turkish tobacco plants (Table 1). In contrast to the results from TMV, both the TRV and PVX virus systems did not show decreases in target *BC-ATPase* mRNA levels after feeding of *B. cockerelli* on leaf discs from Turkish tobacco plant expressing *BC-ATPase* sequence (Table 1). These data suggested that the leaf disc method gave more consistent detectable RNAi effects in *B. cockerelli* compared with whole plant feeding, and TMV was the most promising virus for all of the three plants tested here, among which tomatillo was the best host plant as it was easily infected by TMV and also a most favorable host for *B. cockerelli*.

**Table 1:** Quantitative real-time PCR detection for *B. cockerelli* endogenous *BC-ATPase* mRNAs after feeding on the plants infected with virus expressing *BC-ATPase* interfering sequence.

Plant^1^	Virus^2^	NO. of sample^3^	RNA sample from^4^	Means ± SE inGFP sample^5^	Means ± SE in Test sample^5^	P Value^6^
Tomato	TMV	8	Adult	1±0.26	1.09±0.49	0.680
Tomato	TMV	8	Adult	1±0.23	0.91±0.47	0.656
Tomato	TMV	6	Adult	1±0.19	0.60±0.19	0.007**
Tomato	TMV	5	Adult	1±0.10	0.84±0.20	0.226
Tomato	TMV	7	Nymph	1±0.36	0.86±0.37	0.522
Tomato	TMV	5	Nymph	1±0.14	0.38±0.05	0.001**
Tomato	TMV	5	Nymph	1±0.24	0.24±0.12	0.001**
Tomatillo	TMV	6	Adult	1±0.14	1.13±0.25	0.336
Tomatillo	TMV	6	Adult	1±0.24	1.03±0.28	0.853
Tomatillo	TMV	5	Adult	1±0.09	1.21±0.25	0.151
Tomatillo	TMV	5	Adult	1±0.31	0.54±0.10	0.130
Tomatillo	TMV	6	Adult	1±0.10	1.12±0.15	0.168
Tomatillo	TMV	7	Adult	1±0.40	1.36±0.53	0.209
Tomatillo	TMV	5	Nymph	1±0.49	0.37±0.17	0.043*
Tomatillo	TMV	5	Nymph	1±0.16	0.39±0.08	0.001**
Tomatillo	TMV	5	Nymph	1±0.10	0.64±0.11	0.006**
Tomatillo	TMV	5	Nymph	1±0.78	0.57±0.48	0.376
Tobacco	TMV	5	Nymph	1±0.53	0.73±0.22	0.444
Tobacco	TMV	5	Nymph	1±0.29	0.47±0.35	0.050*
Tobacco	TMV	3	Nymph	1±0.09	0.40±0.06	0.002**
Tobacco	TRV	5	Nymph	1±0.46	1.27±0.41	0.471
Tobacco	TRV	5	Nymph	1±0.63	1.62±0.59	0.261
Tobacco	TRV	5	Nymph	1±0.29	1.38±0.24	0.137
Tobacco	PVX	5	Nymph	1±0.29	1.20±0.37	0.488
Tobacco	PVX	5	Nymph	1±0.40	0.84±0.53	0.697
Tobacco	PVX	5	Nymph	1±0.36	1.10±0.39	0.757

1 The plants used for virus infection and *B. cockerelli* feeding.

2 The virus vector expressing *BC-ATPase* and control GFP sequences for plant inoculation.

3 The number of psyllids used for qRT-PCR analysis in one experiment. For each experiment, the same numbers of GFP samples were used as controls for test sample.

4 Teneral adult *B. cockerelli* or nymphs for feeding experiments and RNA abundance detection were indicated. All of the samples labeled “Nymph” were done using leaf disc method, and the samples labeled “Adult” were done using whole plant feeding experiment.

5 The mRNA abundance of *BC-ATPase* gene after feeding on virus-ATPase –infected plants was shown as test sample, and the average value of the control GFP group was designated as 1. Expression of *BC-ATPase* was normalized to the level of *BC-Actin* in the same sample.

6 Differences between GFP and test group were calculated using the Bonferroni t-test. Single asterisk indicates p<0.05 and double asterisk indicates p<0.01.

### 3. Qualitative and Quantitative Analysis of Ingested RNA in Feeding *B.cockerelli*

**Figure 3:**
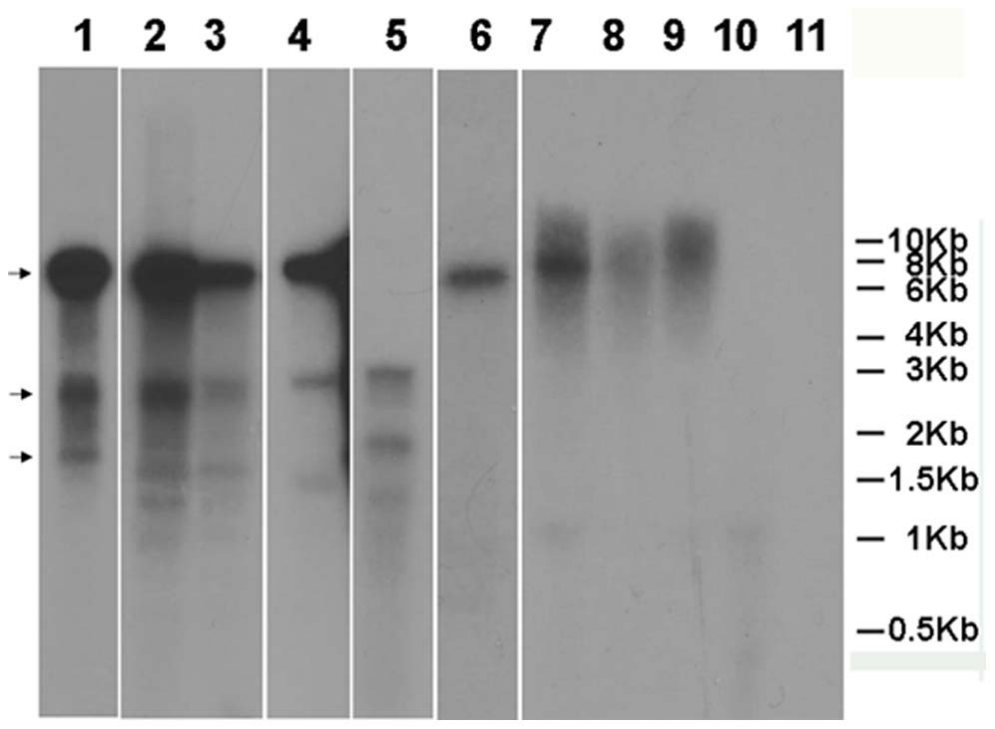
Northern blot hybridization analysis of virus RNAs acquired by nymphal *B.* *cockerelli* after feeding on virus-infected plants. Approximately 60 nymphal *B. cockerelli* were allowed to feed for 24 h on leaf discs from tomatillo, tomato or tobacco plants that were infected with TMV-GFP, PVX-GFP or TRV-GFP. The leaf discs were taken from plants that showed GFP expression under UV light illumination at 14 days post rub-inoculation. Total RNAs from leaf tissues (at 14 days post inoculation) and psyllid nymphs (at 24 h post feeding) were separated by denaturing agarose gel electrophoresis, transferred to nylon membranes, and hybridized with ^32^P-UTP-labeled GFP-specific probes. Lane 1: tomatillo infected with TMV-GFP. Lane 2: tomato infected with TMV-GFP. Lane 3: tobacco plants infected with TMV-GFP. Lane 4: tobacco infected with PVX-GFP. Lane 5: tobacco infected with TRV-GFP. Lane 6: nymphs fed on tomatillo infected with TMV-GFP. Lane 7: nymphs fed on tomato infected with TMV-GFP. Lane 8: nymphs fed on tobacco infected with PVX-GFP. Lane 9: nymphs fed on tobacco infected with TMV-GFP. Lane 10: nymphs fed on tobacco infected with TRV-GFP. Lane 11: control nymphs fed on uninoculated tobacco leaf discs. The migration of size standards (0.5–10 kb RNA ladder) is indicated to the right. The arrows to the left indicate predicted TMV-GFP*-*specific genomic and subgenomic RNAs from top to bottom, respectively.

Plant infections with RNA viruses result in the production of various types of virus-specific RNAs including double-stranded RNAs (replicative forms for+sense ssRNA viruses) [39], and the plants respond to virus infection by generating virus-specific siRNAs [40]. Both of these types of RNAs can induce RNAi effects in *B. cockerelli* and other eukaryotes [8], [28], and at least siRNAs are known to traffic in the phloem [31] where *B. cockerelli* feeds. Thus we sought to identify which forms and how much of the virus RNAs were acquired by *B. cockerelli* during feeding on virus-infected plants. We used TMV-GFP rather than TMV expressing *B. cockerelli* sequences here as the latter might interfere with the data interpretation. First, northern hybridization experiments were performed to qualitatively identify the forms of the ingested virus RNA in *B. cockerelli* after leaf-disc feeding experiments. As shown in Fig. 3, TMV-GFP positive-sense genome-size RNA of ∼7.1 Kb (Black bar) and subgenomic RNAs of ∼2.3 Kb and ∼1.6 Kb (Arrows on the left) were detected in TMV-GFP infected plants (Fig. 3, lanes 1, 2, 3). In PVX-GFP infected tobacco plants, positive-sense genome-size RNA of ∼7.2 Kb and subgenomic RNAs of ∼2.8 Kb and ∼1.7 Kb were detected (Fig. 3, lane 4). In TRV-GFP infected tobacco plants, positive-sense TRV RNA 2 genome-size RNA of ∼3 Kb and subgenomic RNAs of ∼2.0 Kb and ∼1.5 Kb were detected (Fig. 3, lane 5). When we analyzed *B. cockerelli* which fed on leaf discs from these plants we were only able to consistently identify positive-sense genome-size RNAs for TMV-GFP and PVX-GFP (Fig. 3, lane 6, 7, 8, 9), but not in TRV-GFP fed *B. cockerelli* (Fig. 3, lane 10).

**Figure 4:**
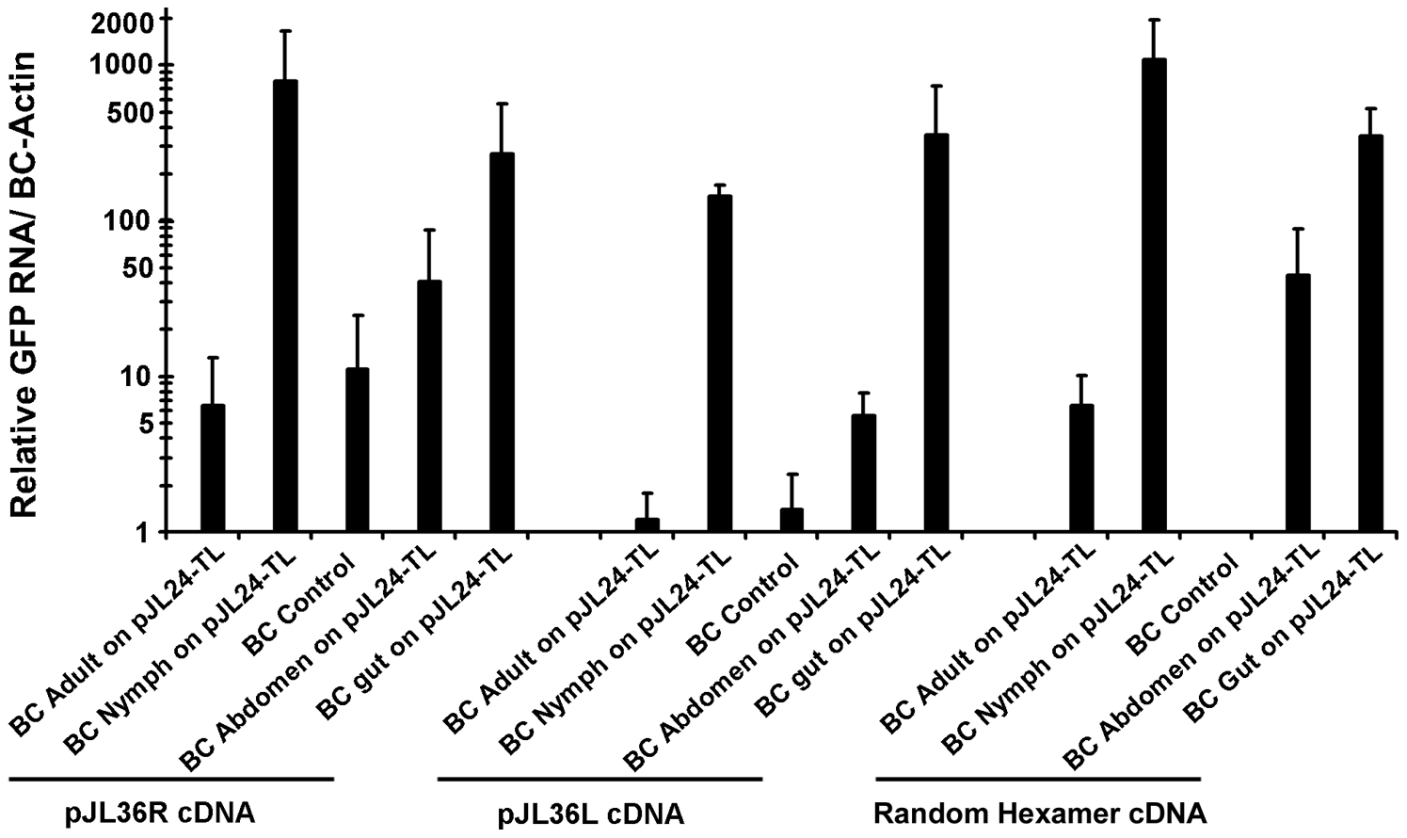
Quantitative real-time PCR analysis of the oral acquisition of virus RNAs by *B. cockerelli*. Teneral adults and nymphs were allowed to feed on whole plants or leaf discs, respectively, of TMV-GFP-infected tomatillo plants (14 days post inoculation) for 24 h. Following feeding, total RNAs were isolated from whole adults, adult abdomens, adult guts, and whole nymphs. cDNAs were generated using primers that were specific for positive sense (pJL36R) or negative sense (pJL36L) TMV-GFP RNA, or random hexamer primers. Quantification of the amount of TMV-GFP RNA that was acquired by *B. cockerelli* was performed with *GFP*-specific qRT-PCR and was normalized against constitutively-expressed psyllid *BC-Actin* mRNA. BC is the abbreviation for *B. cockerelli*, and pJL24-indicates the pJL24 (TMV-GFP)-infected tomatillo plants. We did obtain consistent positive signals for GFP RNA in the BC control samples when using pJL36R cDNA. By contrast, both pJL36L and Random Hexamer cDNAs showed essentially zero for the BC controls, thus supporting our interpretation of these data.

We next compared the quantity of RNAs acquired by *B. cockerelli* after feeding on TMV-GFP-infected tomatillo plants. qRT-PCR analyses showed that *B. cockerelli* nymphs consistently showed >100 times more TMV-GFP RNA in comparison to the teneral adult *B. cockerelli* (Fig. 4). We also dissected adult *B. cockerelli* and compared RNA amounts from pooled guts and abdomens and consistently found 10 times or greater TMV-GFP RNAs from isolated guts compared to the abdomen samples. We performed qRT-PCR separately for both TMV-GFP positive- and negative-strand RNAs in order to see if both were acquired by feeding *B. cockerelli*. qRT-PCR results showed detection of both positive and negative-strand TMV RNAs in all samples. In general, more positive-strand TMV-GFP RNA was detected in all samples except for *B. cockerelli* dissected gut tissues (Fig. 4). The above data clearly showed that *B. cockerelli* acquired virus RNAs from plants.

### 4. RNAi Effects on Target mRNAs are Predominantly Observed in Gut Tissues

**Figure 5:**
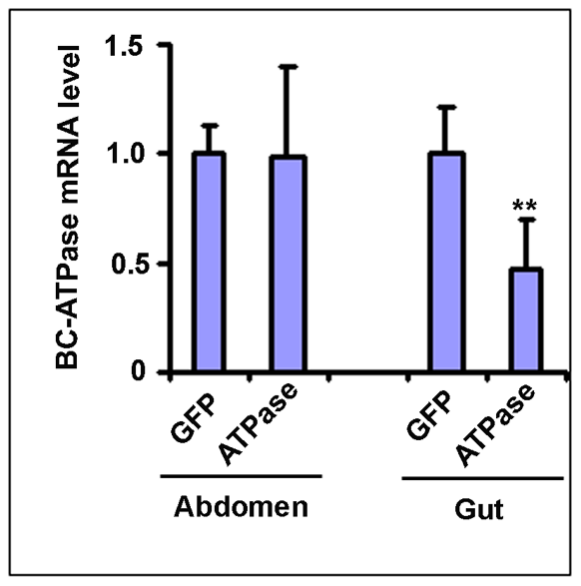
Quantitative real-time PCR analysis of *BC-ATPase* mRNAs in *B.* *cockerelli* whole insects and specific tissues. Teneral adults were allowed to feed for 72 h on tomatillo plants that were infected (14 days post inoculation) with TMV-ATPase or TMV-GFP. Following feeding, total RNAs were isolated from the abdomen or gut tissues of ∼100 insects for each treatment. The experiments were repeated three times. cDNAs were generated using random hexamer primers, and the cDNA was used for quantitative real-time PCR using *BC-ATPase* specific primers. The qRT-PCR results were normalized against constitutively expressed *BC-Actin* levels. The level of *BC-ATPase* mRNA in psyllids that fed on TMV-GFP-infected plants was arbitrarily designated as 1. Differences between *BC-ATPase* mRNA levels in *B. cockerelli* fed on TMV-ATPase-infected and control TMV-GFP-infected plants were analyzed using the Bonferroni *t-test*. The double asterisks indicate a significant difference at p<0.01. Gut GFP (t = 6.831, df = 11), Gut ATPase (t = 15.660, df = 11); Abdomen GFP (t = 5.889, df = 6), Abdomen ATPase (t = 19.265, df = 6).

**Figure 6:**
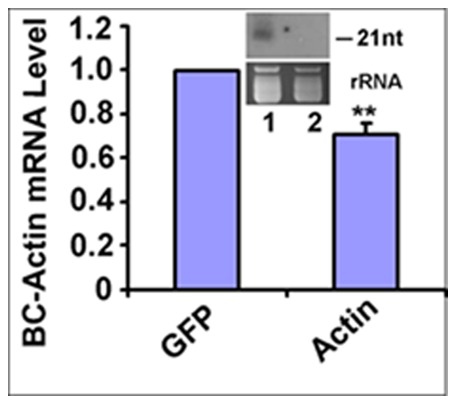
Quantitative real-time PCR analysis of *actin* mRNA levels in gut tissues of *B.* *cockerelli*. Teneral adults were allowed to feed for 72 h on tomatillo plants that were infected (14 days post inoculation) with TMV-actin or TMV-GFP. Following feeding, total RNAs were isolated from the gut tissues of ∼100 *B. cockerelli*. The experiments were performed for three biological repeats. cDNAs were generated using random hexamer primers, and the cDNA was used for quantitative real-time PCR using *actin-*specific primers. The qRT-PCR results were normalized to the level of rRNA of *B. cockerelli*. Differences between *actin* mRNA levels in *B. cockerelli* that fed on TMV-actin-infected and control TMV-GFP-infected plants were analyzed using the Student *t-test*. Double asterisks indicate p<0.01. GFP (t = 19.776, df = 2), Actin (t = 19.347, df = 2). Inset: The inset panel shows ∼21 nt- siRNAs that were detected in TMV-Actin-infected plants at 14 days post inoculation by Northern hybridization using an *actin* specific probe. The lower part of the panel shows an rRNA loading control. Lane 1, TMV-Actin inoculated tomatillo, lane 2, Untreated tomatillo control.

As the *BC-ATPase* mRNA is primarily highly expressed in gut tissues [28], we then aimed to understand tissue-specific RNAi effects on *BC-ATPase* mRNAs after feeding on TMV-ATPase-infected tomatillo plants. As it is very difficult to dissect and isolate tissues from *B. cockerelli* nymphs, teneral adults were used for these experiments. Teneral adults were allowed to feed on the lower expanded leaves of TMV-ATPase- and TMV-GFP-infected tomatillo plants for 3 days, and the total RNA was prepared from dissected gut and abdomen tissues as a pooled sample from ∼100 *B. cockerelli* and used for *BC-ATPase* mRNA quantification. These analyses showed that the *BC-ATPase* mRNA abundance was decreased by ∼50% in gut tissues but no reduction was detected in abdomen tissues (Fig. 5), consistent with our previous data using artificial diet feeding assays [28]. These data were also consistent with that above showing that virus RNAs were acquired by and primarily detected in gut tissues of teneral adults (Fig. 4). As *BC-Actin* is globally expressed and RNA interference was only detected in gut tissues in dsRNA feeding experiments [28], we then examined RNAi effects on this target mRNA after feeding on TMV-Actin infected tomatillo plants. TMV-Actin infected tomatillo accumulated *BC-Actin* siRNAs and *B. cockerelli* fed on the plants showed a reduced mRNA abundance of ∼70% in their gut tissues (Fig. 6).

### 5. Recombinant TMVs for *BC-ATPase* and *BC-Actin* Reduce *B. cockerelli* Fecundity

**Figure 7:**
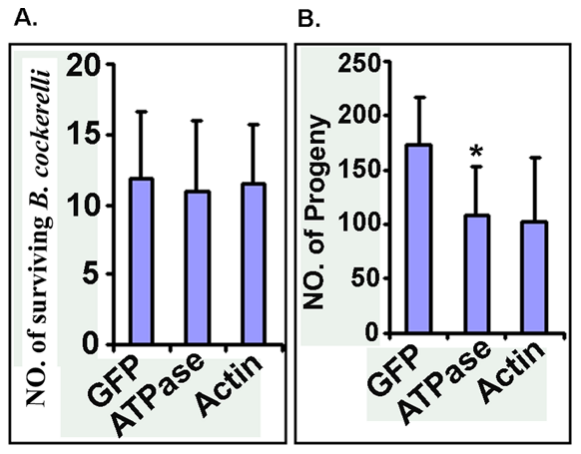
RNAi effects targeting *ATPase* and *actin* mRNAs and survival and fecundity of *B.* *cockerelli*. Teneral adults (groups of 20) were allowed to feed for 7 days on tomatillo plants that were inoculated with TMV-GFP, TMV-ATPase and TMV-Actin (10 days post inoculation). After 7 days of feeding on the virus-infected tomatillo, all of the adult psyllids were removed and numbers of survivors was determined. The tomatillo plants (without any adults) were then returned to the growth chamber for another 14 days and the number of progeny nymphs was assessed. The experiments were repeated three times and five biological replicates were included in each experiment. Differences in survival and fecundity between the insects that fed on TMV-ATPase- or TMV-actin-infected tomatillo and control (TMV-GFP-infected) plants were analyzed using the Bonferronit-test. The asterisk indicates a significance at p<0.01. (A), Number of surviving *B. cockerelli* after feeding for 7 days on virus-infected tomatillo; GFP (T = 14.736, df = 4), ATPase (t = 12.863, df = 4), Actin (T = 14.969, df = 4). (B), number of progeny nymphs at 14 days after removal of the adult psyllids; GFP (T = 7.8, df = 4), ATPase (T = 4.935, df = 4), Actin (T = 3.519, df = 4).

We previously showed that *B. cockerelli* feeding on artificial diets containing specific dsRNAs, including those for *BC-ATPase* and *BC-Actin,* exhibited increased mortality for *B. cockerelli* in addition to mRNA reductions [28]. Hence, we next assessed if similar effects on the survival of *B. cockerelli* could be induced by plant virus-mediated delivery of *BC-ATPase* or *BC*-*Actin* interfering RNAs. Twenty teneral adult *B. cockerelli* were fed on respective virus-infected tomatillo plants, and were collected after 7 days. The numbers of surviving *B. cockerelli* on TMV-GFP, TMV-ATPase and TMV-Actin infected plants were 11.8±4.8, 10.9±5.1 and 11.5±4.1, respectively, showing no differences in survival (Fig. 7 ). However, when the plants were maintained for an additional 14 days and when the progeny on these plants were counted, the numbers for the TMV-GFP, TMV-ATPase and TMV-Actin infected plants were 173±44, 108±44 and 103±58, respectively (Fig. 7). Thus, nymph numbers for the TMV-ATPase group were decreased by ∼38% (P value = 0.0134) and for the TMV-Actin group by ∼40% (P value = 0.0537) when compared to the TMV-GFP group (Fig. 7).

## Discussion

Several groups have demonstrated successful insect-targeted RNAi effects by using stable transgenic plants engineered to express RNAi inducers, and recently *Agrobacterium tumefaciens*-mediated delivery of RNAi inducer sequences into leaves of non-transgenic plants has shown success [18]–[21], [41]. The time, labor and cost required for generation of transgenic plants precludes efficient large-scale assays to assess RNAi effectors *in planta,* and while assays using *A. tumefaciens* transient expression offer a rapid approach, plant virus-based expression provides additional benefits including RNA amplification during virus replication and generation of dsRNA and siRNA inducers, most likely resulting in a longer, systemic and higher expression for the insert sequences. In this regard, use of recombinant *Tobacco rattle virus* was recently used by Kumar et al. to induce RNAi effects towards the leaf feeding lepidopteran, *Manduca sexta* [25]. *M. sexta* is a voracious feeder ingesting all leaf tissues and readily consumes virus-infected cells. By contrast, here we demonstrated plant virus-mediated RNAi induction against the phloem-feeding hemipteran, *B. cockerelli*.

Previous efforts have demonstrated that RNAi effects can be induced against phloem feeding hemipterans when they feed on transgenic plants [21], or even by transient leaf disc feeding assays [20]. To induce RNAi effects towards phloem feeders, effector RNAs must be available for phloem transport, and then acquired by and induce effects on recipient insects. Plant viruses thus offer a unique opportunity here, as almost all plant-infecting viruses utilize phloem tissues for long-distance (systemic) transport and spread within plants. Furthermore, RNA virus infection results in production of at least two types of RNAs that are capable of acting as RNAi effectors. These are double-stranded RNAs produced during RNA virus replication [39] and siRNAs, produced as part of the plants anti-viral defense activity [40]. Of these, at least siRNAs have been recovered from plant phloem [31]. Therefore, it seemed reasonable to us to test for RNAi effects against the phloem-feeding *B. cockerelli* by using recombinant RNA virus infection assays.

We tested here various virus:plant combinations for the ability to induce RNAi effects against *B. cockerelli. B. cockerelli* has a relatively broad host range, and tomato, tomatillo and Turkish tobacco plants were the best hosts here for our *B. cockerelli* virus studies. TMV infected all of these three plants and sequence-specific ∼21nt siRNAs were easily detected from the TMV-infected plants. Most importantly, we were able to induce RNAi effects towards the target RNAs when *B. cockerelli* fed on these plants. Compared with TMV, expression of interfering sequences with both TRV and PVX did not cause significant RNAi effects on recipient *B. cockerelli*.

We evaluated different feeding methods and found that feeding *B. cockerelli* nymphs on leaf discs to be much more efficient and consistent for assessing RNAi effects than was feeding teneral adult *B. cockerelli* on whole plants. Two possible explanations for this are: first, that the lower expanded leaves are source leaves, active in phloem transport activity and virus replication, thereby expressing higher levels of recombinant virus RNAs including siRNAs. Second, *B. cockerelli* nymphs are more active feeders than the teneral adult *B. cockerelli.* We used quantitative real-time PCR and found that *B. cockerelli* nymphs acquired more than 100 times of virus sequences than did the teneral adult *B. cockerelli*. We do not know whether one or more types of virus-specific RNAs induce RNAi effects in *B. cockerelli.* Virus-specific ssRNAs, dsRNAs and siRNAs are produced during virus infections of the plant, and many of the progeny ssRNAs are encapsidated within newly assembled virus particles. Both dsRNAs and siRNAs are well known RNAi inducers, and siRNAs are known to traffic in the plant phloem [31]. We used northern hybridization and real time RT-PCR assays to identify virus-specific RNAs in *B. cockerelli* and northern hybridization assays showed that *B. cockerelli* readily acquired positive-sense virus RNAs. However, our real time RT-PCR assays indicated the presence of both virus positive and negative strands in *B. cockerelli*, suggesting that *B. cockerelli* may be acquiring dsRNAs from plants. We also attempted to detect siRNAs in *B. cockerelli* fed on TMV-ATPase-infected plants, but like our previous studies when we used artificial diets to deliver dsRNAs [28] this assay proved to be not sufficiently sensitive (data not shown). Whether the virus-specific siRNAs are abundant in the phloem and may serve as a source for inducing RNAi effects in recipient *B. cockerelli* remains to be determined.

We were able to demonstrate statistically significant reduction of *BC-ATPase* mRNA levels in *B. cockerelli* gut tissues but not in abdomen tissues after feeding on TMV-ATPase-infected plants. These results most likely reflect that the midgut is one of the first barriers that ingested effector RNAs will encounter following feeding, and our previous data using artificial diet dsRNA feeding experiments, as well as those for other insects, show that midgut-expressed mRNAs are susceptible to the effects of orally-delivered dsRNAs [8], [16], [19], [21]. It is important to note that RNAi effects towards *BC-ATPase* and also *BC-Actin* mRNAs did not influence the survival of feeding tenereal *B. cockerelli* in our experiments. However, we did find a decrease in the number of progeny *B. cockerelli* that developed on plants infected with the viruses. These results are consistent with data for another phloem feeding Hemipteran, *Myzus persicae*, where the aphids survived equally well but produced fewer nymphs on MpC002 or Rac-1 silenced hairpin-expressing transgenic *Arabidopsis* plants [20]. Along the same lines, it is also interesting to note that *B. cockerelli* survival from egg to adult is higher on susceptible tomato cultivar, Moneymaker (mi-1.2) than that on the resistant cultivar, Motelle (Mi-1.2), whereas no effects were observed for adult survival and oviposition rate [42]. Furthermore, in *Tribolium casteneum*, injection of double-stranded RNA (dsRNA) into the parent insects resulted in RNAi effects towards zygotic mRNA in offspring embryos [10]. Therefore, it seems reasonable to speculate that progeny, or nymphal insects may be more vulnerable to the resistance genes (mi-1.2 for *B. cockerelli*) and/or RNAi effects (our data and *T. casteneum*) than are adult insects. Our data show that *B. cockerelli* nymphs acquire 100 times more RNAs from the plant, thus dose effects could also have a role in gene silencing efficiency. It is also important to note that the effects on adult *B. cockerelli* survival were moderate here in comparison to dsRNA artificial diet feeding assays [28], and thus if RNAi assays only assess mortality they could be misleading. In conclusion, we demonstrated the induction of RNAi effects in the potato/tomato psyllid (*B. cockerelli*) via plant virus expression of specific sequences. This demonstrates the possibility of inducing RNA interference via plant expression for phloem feeders like *B. cockerelli* and can provide a rapid and high throughput alternative assay as opposed to transgenic approaches. Furthermore, recombinant plant-infecting viruses might even have potential for developing new approaches for RNAi-mediated insect control strategies, particularly on perennial hosts like woody plants where target insects may reproduce.

## Supporting Information

Figure S1 **Illustration of the different feeding methods used here.** (a), Whole plant feeding of teneral adult *B. cockerelli* using plastic cylinder cages; (b), Mesh cage feeding of teneral adult *B. cockerelli* on lower expanded leaves; (c), Leaf disc feeding method using *B. cockerelli* nymphs. Fourteen days after inoculation leaf discs were harvested from respective leaves using a cork borer. Leaf discs were placed upside-down on MS agar in a 12-well plate and psyllid nymphs were fed on the disks.
(PDF)
