# Supplementary figures and images for: RNA Interference towards the Potato Psyllid, Bactericera cockerelli, Is Induced in Plants Infected with Recombinant Tobacco mosaic virus (TMV)
Source: PLoS One. 2013 Jun 18;8(6):e66050. doi: 10.1371/journal.pone.0066050 (PMC3688868; doi:10.1371/journal.pone.0066050)

# Supplementary Figure 1

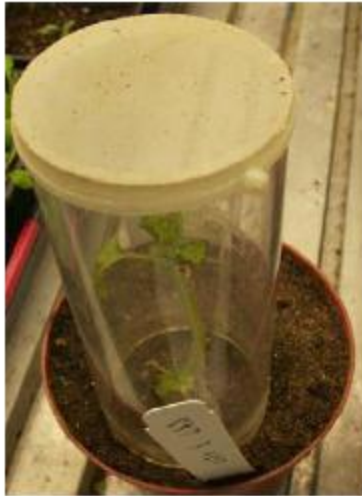

(a)

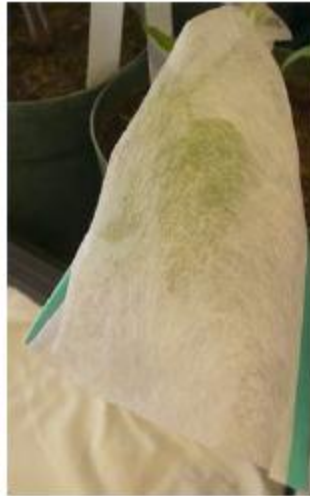

(b)

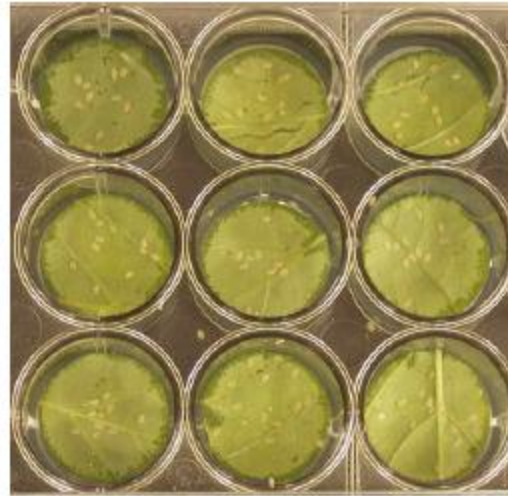

(c)

Supplement: Figure S1 — Illustration of the different feeding methods used here. (a), Whole plant feeding of teneral adult B. cockerelli using plastic cylinder cages; (b), Mesh cage feeding of teneral adult B. cockerelli on lower expanded leaves; (c), Leaf disc feeding method using B. cockerelli nymphs. Fourteen days after inoculation leaf discs were harvested from respective leaves using a cork borer. Leaf discs were placed upside-down on MS agar in a 12-well plate and psyllid nymphs were fed on the disks. (PDF) [file pone.0066050.s001.pdf]
